# Effects of exercise habits in adolescence and older age on sarcopenia risk in older adults: the Bunkyo Health Study

**DOI:** 10.1002/jcsm.13218

**Published:** 2023-04-13

**Authors:** Hiroki Tabata, Hikaru Otsuka, Huicong Shi, Mari Sugimoto, Hideyoshi Kaga, Yuki Someya, Hitoshi Naito, Naoaki Ito, Abulaiti Abudurezake, Futaba Umemura, Mai Kiya, Tsubasa Tajima, Saori Kakehi, Yasuyo Yoshizawa, Ryuzo Kawamori, Hirotaka Watada, Yoshifumi Tamura

**Affiliations:** ^1^ Sportology Center Juntendo University Graduate School of Medicine Tokyo Japan; ^2^ Department of Sports Medicine & Sportology Juntendo University Graduate School of Medicine Tokyo Japan; ^3^ Department of Metabolism & Endocrinology Juntendo University Graduate School of Medicine Tokyo Japan; ^4^ Juntendo University Graduate School of Health and Sports Science Chiba Japan; ^5^ Center for Healthy Life Expectancy Juntendo University Graduate School of Medicine Tokyo Japan; ^6^ Faculty of International Liberal Arts Juntendo University Tokyo Japan

**Keywords:** Adolescence, Older age, Exercise habits, Muscle function, Older adults, Sarcopenia

## Abstract

**Background:**

Sarcopenia, defined as an age‐associated loss of skeletal muscle mass and function, is a major risk factor for requiring long‐term care. Because physical activity in adolescence and older age enhances peak muscle function in youth and prevents muscle function decline in older age, older adults with exercise habits during both periods may be at a lower risk for sarcopenia. We investigated the relationship between exercise habits in adolescence and older age and sarcopenia and its components in community‐dwelling older Japanese adults.

**Methods:**

This study included 1607 community‐dwelling individuals (aged 65–84, medians 73 years, 679 men and 928 women) with complete health examinations, including measurements of skeletal muscle index, handgrip strength and gait speed, who were enrolled in the Bunkyo Health Study. We divided the participants into four groups according to exercise habits in adolescence and older age: no exercise in either period (none‐none; NN), exercise only in adolescence (active‐none; AN), exercise only in older age (none‐active; NA) and exercise in both periods (active‐active; AA). Multivariate‐adjusted logistic regression models were used to estimate the odds ratios (ORs) and associated 95% confidence intervals (CIs) in each group for the prevalence of sarcopenia, defined as low muscle mass and low muscle performance, as compared with the NN group. Low muscle performance was defined as low muscle strength and/or low gait speed.

**Results:**

The total prevalence of sarcopenia was 6.6% (45/679) in men and 1.7% (16/928) in women, the total prevalence of low muscle mass was 14.3% (97/679) in men and 5.2% (48/928) in women, and the total prevalence of low muscle performance was 25.6% (174/679) in men and 19.6% (182/928) in women. In men, the ORs (95% CIs) for sarcopenia, low muscle mass and low muscle performance were significantly lower in the AA group (sarcopenia: 0.29 [0.09–0.95], *P* = 0.041; low muscle mass: 0.21 [0.09–0.52], *P* = 0.001; and low muscle performance: 0.52 [0.28–0.97], *P* = 0.038). In women, the OR (95% CI) for low muscle performance was significantly lower in the AA group than in the other groups (0.48 [0.27–0.84], *P* = 0.010), whereas none of the ORs for sarcopenia and low muscle mass were significant.

**Conclusions:**

Older men with exercise habits in both adolescence and older age were at a lower risk of sarcopenia, low muscle mass and low muscle performance, whereas older women with exercise habits at both time periods were at a lower risk of low muscle performance.

## Introduction

The number of older adults, that is, persons aged ≥65 years, has increased worldwide, and the ageing rate in Japan is the highest in the world.^1^ A serious social problem in an aged society is the increase in the number of older adults requiring long‐term care. The number of Japanese individuals requiring long‐term care reached 6.06 million in 2018,^2^ and increasing continues. One of the major risk factors for long‐term care is sarcopenia,^3^ defined as a decrease in skeletal muscle mass, strength and physical function.^4^ A recent meta‐analysis showed that low muscle mass, low muscle strength and low physical performance were associated with declines in activities of daily living and instrumental activities of daily living in older adults.^5^ We note that Asians have a relatively low body mass index (BMI) compared with that of other ethnicities and that a low BMI is a strong predictor of sarcopenia in these populations.^6^, ^7^ Therefore, sarcopenia could easily develop in older Asian individuals.^8^ Thus, preventing sarcopenia is an important strategy for preventing disability and the requirement for long‐term care and may be especially important in Asian populations.

Previous studies have suggested that current and past exercise habits are associated with skeletal muscle function and are preventive for sarcopenia. For example, in a study conducted on Japanese women, physical activity in midlife and older age was associated with a higher muscle mass and higher walking speed, respectively, in older age.^9^ Exercise habits in middle age were associated with a lower odds ratios (ORs) for sarcopenia in old age in another Japanese study,^10^ and higher leisure‐time physical activity levels across midlife were positively associated with handgrip strength at age 60–64 years in previous research.^11^ Considering the fact that muscle function is enhanced in adolescence and is gradually impaired with older age,^12^ we hypothesized that older adults who exercise in both adolescence and older age might exhibit higher muscle function than those who did not exercise in either period. To our knowledge, only one study has tested this hypothesis to date. Specifically, a previous study suggested that older Japanese women who exercise currently and in adolescence have higher muscle strength than those who did not exercise in both periods.^13^ However, this previous study included only women. Most importantly, it remains unknown whether a combination of exercise habits in adolescence and older age is associated with a lower risk of sarcopenia.

According to this context, the present study aimed to investigate the associations between exercise habits in adolescence and older age and sarcopenia and its components in community‐dwelling older Japanese adults. Our study provides insights into whether lifetime exercise habits can prevent sarcopenia.

## Methods

### Study design and participants

This cross‐sectional research used baseline data from the Bunkyo Health Study, a cohort study investigating associations between muscle mass and muscle strength and insulin sensitivity regarding the main causes of and risk factors for needing long‐term care.^14^ This study recruited older individuals aged 65–84 years who were living in Bunkyo‐Ku, an urban area in Tokyo, Japan; 1629 participants completed the 2‐day study examination at the Sportology Center between 15 October 2015 and 1 October 2018. Briefly, participants were evaluated using physical fitness tests (dynamometry), physical performance tests, brain lesion evaluations [magnetic resonance imaging (MRI)], body composition and bone mineral density (BMD) testing [dual‐energy X‐ray absorptiometry (DXA)], arteriosclerosis (cardio‐ankle vascular index) and abdominal fat distribution (MRI).

**Figure 1:**
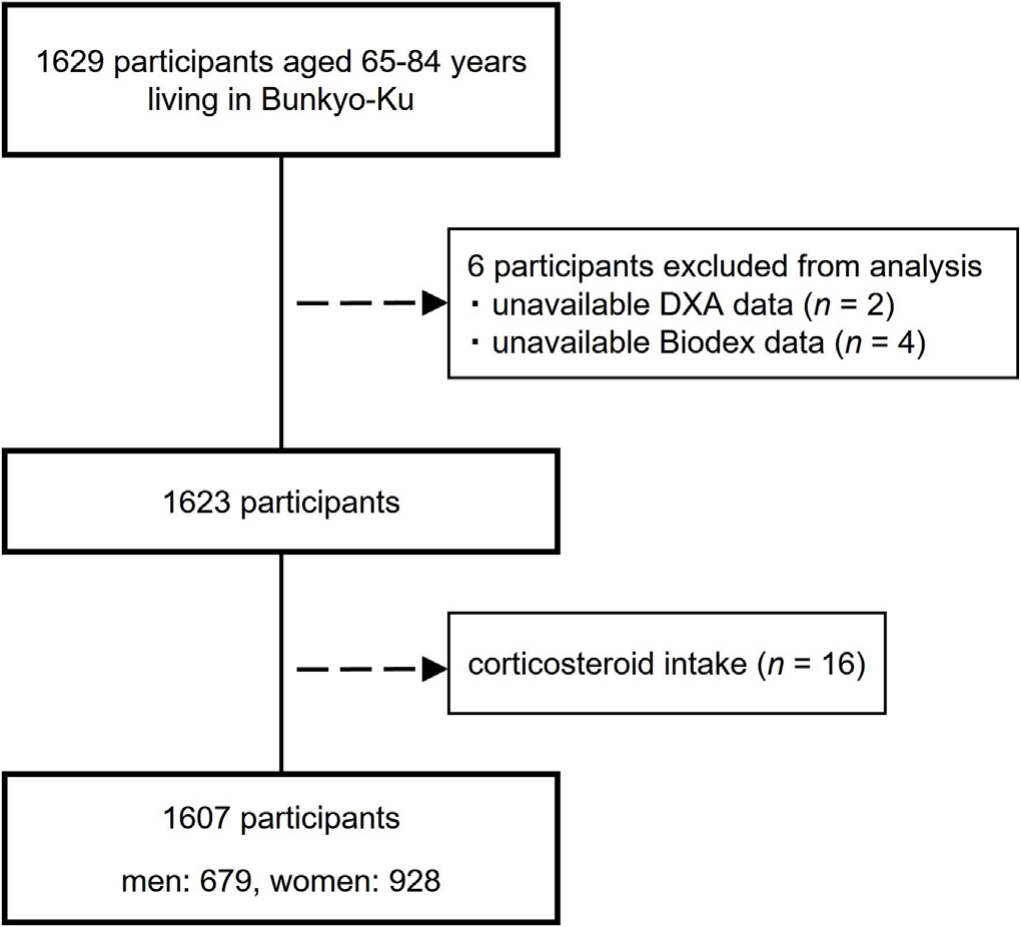
Flowchart of the participants enrolment.

The study protocol was approved by the ethics committee of Juntendo University in November 2015 (first approval no. 2015078 and the latest revised version no. 2021095). This research was conducted in accordance with the principles of the Declaration of Helsinki. All participants provided their written informed consent and were notified that they had the right to withdraw from the trial at any time without incurring any adverse consequences. Several exclusion criteria were established in this study in order to ensure an appropriate and valid evaluation. Specifically, of the 1629 participants enrolled in the Bunkyo Health Study, the current study excluded six participants with unavailable data (DXA [*n* = 2], lower limb isokinetic muscle strength [*n* = 4]). Of the remaining 1623 participants, 16 participants receiving corticosteroids were excluded as well because corticosteroids induced muscle atrophy. Finally, a total of 1607 participants (male: 679, female: 928) were included in the present analysis (*Figure* 1).

### Assessment of exercise habits

All participants were interviewed using the following questions: ‘Did you participate in sports club activities when you were in junior high school or high school?’ and ‘Do you currently have exercise habits?’ If they answered ‘yes’, we asked about engagement in specific sports, club activities, current exercise types and frequency. We showed the types of sports club activities in junior high school or high school and currently in old age in which participants engaged in *Tables* S1 and S2. Additionally, current physical activity levels were evaluated using the International Physical Activity Questionnaire.^15^, ^16^ We defined those who had participated in sports club activities during junior high school or high school as having exercise habits during adolescence. Based on the definition of exercise habits in the National Health and Nutrition Exercise Survey in Japan,^17^ those who exercised more than twice a week for at least 30 min per session currently were defined as having exercise habits at present, that is, in older age. Also, we asked, ‘Did you have exercise habits at each age from your 20s to 50s?’, those who responded ‘yes’ were defined as having exercise habits at each age from their 20s to 50s.

### Measurement of skeletal muscle function

Skeletal muscle mass was measured using DXA (Discovery DXA System, Hologic, Tokyo, Japan).^18^ The skeletal muscle mass index (SMI) was calculated by dividing muscle mass by height in meters squared (kg/m^2^). Lower limb isokinetic muscle strength was measured using a dynamometer (BIODEX system 3 or 4: Biodex Medical Systems, Upton, NY, USA).^19^ Participants were stabilized in the examination chair using shoulder and abdominal straps, and the isokinetic peak torques of the knee extensors were measured at an angular velocity of 60°/s. During the test, the participants were encouraged to exert maximal muscle force. The isokinetic peak torques of the knee extensors were adjusted for body weight according to the following formula: isokinetic peak torques (Nm)/body weight (kg).

We evaluated skeletal muscle strength based on handgrip strength and evaluated physical performance based on maximum gait speed. Handgrip strength was measured using a handgrip dynamometer (T.K.K. 5401, Takei Scientific Instruments, Niigata, Japan) with the arm held to the side of the body.^20^ The participants squeezed the dynamometer with maximum isometric effort. No other body movement was allowed. Two trial measurements were recorded alternately for each hand, and the average of the better values for each hand was calculated and adopted. The maximum gait speed was tested twice in the middle 5 m of the 10‐m course. Using the better walking time of the two trials, gait walking speed (m/s) was calculated to obtain values for analysis.

We also measured several myokines, including myostatin, brain‐derived neurotrophic factor (BDNF), osteonectin and fatty acid‐binding protein (FABP). Myokines are an essential indicator in evaluating muscle function; for example, myostatin decreases muscle mass and induces muscle atrophy, whereas fibroblast growth factor 21 (FGF‐21) improves muscle mass and muscle mitochondrial function.^21^ Serum myostatin and serum fibroblast growth factor 21 (FGF‐21) levels were measured using an enzyme‐linked immunosorbent assay (GDF‐8/Myostatin Quantikine ELISA Kit and Human FGF‐21 Quantikine ELISA Kit, R&D Systems, Minneapolis, MN, USA). Serum BDNF, serum osteonectin and serum FABP3 levels were measured using a multiplex assay (MILLIPLEX MAP Human Myokine Magnetic Bead Panel, Merck, Darmstadt, Germany).

### Definition of sarcopenia

We used a modified algorithm provided by the Asian Working Group of Sarcopenia (AWGS) 2019 to define sarcopenia according to muscle mass, muscle strength and physical performance.^22^ Low muscle mass was defined as an SMI of <7.0 kg/m^2^ for men and <5.4 kg/m^2^ for women. Low muscle strength was defined as a handgrip strength of <28.0 kg for men and <18.0 kg for women. Low physical performance was defined as a maximum gait speed of <1.46 m/s for men and <1.36 m/s for women. Because we did not measure the Usual gait speed in the present study, we used cut‐off estimation values for maximum gait speed that corresponded to the usual gait speed, which was calculated using gender‐specific linear regression.^23^ Sarcopenia was defined as low muscle mass and low muscle performance (i.e. low muscle strength and/or low physical performance).

### Other measurements

A physician recorded medical history and information on current medications in a semi‐structured format. Self‐administered questionnaires were employed to determine the following: sex (male or female), age (years) and smoking status (current and former smoking). Blood samples were taken in the morning after an overnight fast to perform appropriate biochemical tests. Blood glucose and haemoglobin A1c levels were tested at the commissioned clinical laboratory centre (SRL Inc., Tokyo, Japan). Diabetes mellitus was defined as a fasting plasma glucose level of ≥126 mg/dL and/or a 2‐h glucose level of ≥200 mg/dL following a 75 g oral glucose tolerance test, a haemoglobin A1c level of ≥6.5% or currently taking medication for diabetes mellitus. The BMD of the hip joint (total hip) and lumbar spine (L2–L4) was measured using DXA (Discovery DXA System; Hologic Inc, Marlborough, Massachusetts, USA) and expressed as standard deviation (SD) units relative to the BMDs of young persons (T‐score). Osteoporosis was defined as a BMD T‐score of −2.5 or less for lumbar spine or hip bone density based on World Health Organization criteria or currently taking medication for osteoporosis.^24^, ^25^ Dietary intake was assessed using a brief self‐administered diet history questionnaire (BDHQ) to measure protein intake. The BDHQ had been validated in previous studies.^26^

### Statistical analysis

Participants were divided into four groups according to exercise habits as follows: those who (i) never exercised (none‐none; NN), (ii) exercised only in adolescence (active‐none; AN), (iii) exercised only in older age (none‐active; NA) and (iv) exercised both in adolescence and older age (active‐active; AA). Participant characteristics were compared using Kruskal–Wallis and chi‐squared tests for continuous and categorical variables, respectively. Continuous variables were reported as medians (interquartile ranges), whereas categorical variables were indicated as frequencies (percentages). Logistic regression models were used to measure ORs and associated 95% confidence intervals (CIs) for the prevalence of sarcopenia, low muscle mass and low muscle performance in the AN, NA and AA groups as compared with the NN group (i.e. the referent). Model 1 was adjusted for age (continuous) and BMI (continuous). Model 2 was adjusted for the Model 1 covariates as well as years of education (continuous), current and past smoking status (yes or no) and protein intake (continuous). Model 3 was adjusted for the Model 2 covariates as well as current diabetes mellitus (yes or no), current cardiovascular diseases (yes or no), including cerebrovascular diseases and current osteoporosis (yes or no). Model 4 was adjusted for the Model 3 covariates as well as the young adult to middle‐age exercise habits score. The young adult to middle‐age exercise habits score was calculated according to having exercise habits in the 20s–50s: score plus 1 for having an exercise habit and 0 for not having an exercise habit of each age period. Skeletal muscle function differences among the four study groups were compared using analysis of covariance (ANCOVA) adjusted for the following potential confounders: age, years of education, smoking status, protein intake and presence of diabetes mellitus, current cardiovascular diseases and current osteoporosis. We adjusted for multiple comparisons using *post hoc* Bonferroni correction. Values were presented as means ± standard errors. Statistical analyses were performed using SPSS statistical software (ver. 28.1 for Windows; IBM Corporation, Armonk, NY, USA). A *P*‐value of <0.05 was considered statistically significant.

## Results

### Participant characteristics

Participant characteristics according to exercise habits in adolescence and older age in both men and women are presented in *Table* 1. In men, age was significantly younger in the AA group than in the NN group, and the body fat percentage was significantly lower in the AA group than in the other groups. Physical activity levels were approximately two times higher in the NA and AA groups than in the AN and NN groups, reflecting current exercise habits. In women, height was significantly taller in the AA group than in the NN group. The prevalence of osteoporosis was lower in the AA group than other three groups. Similar to findings in men, physical activity levels were approximately 1.5 times higher in the NA and AA groups than in the AN and NN groups.

**Table 1:** Participant medical and demographic characteristics by sex and exercise group

Men (*N* = 679)	None‐None (*N* = 131)	None‐Active (*N* = 121)	Active‐None (*N* = 257)	Active‐Active (*N* = 170)	*P* value
Age (years)	74 (69–79)	73 (69–78)	72 (68–77)	72 (68–75)^†^	0.006
Height (cm)	164.8 (160.9–168.2)	165.5 (160.7–169.4)	166.0 (162.0–170.2)	166.5 (161.5–170.9)	0.071
Body mass (kg)	64.9 (59.6–69.7)	62.3 (56.9–68.2)	65.0 (59.3–70.6)	63.9 (58.2–71.1)	0.174
Body mass index (kg/m^2^)	23.8 (22.2–25.3)	23.1 (21.2–25.0)	23.4 (21.8–25.0)	23.1 (21.6–25.1)	0.068
% body fat (%)	19.1 (16.8–22.6)	17.7 (15.2–21.6)	18.0 (15.1–20.7)	16.8 (14.6–19.2)^†^ ^,^ ^#^ ^,^ ^§^	<0.001
Fat‐free mass (kg)	51.9 (47.9–56.9)	50.7 (47.2–55.6)	53.0 (48.1–57.8)	53.0 (48.9–57.5)	0.039
Education years (years)	16.0 (12.0–16.0)	16.0 (13.5–16.0)	16.0 (12.0–16.0)	16.0 (16.0–16.0)^§^	0.009
Protein intake (g/day)	76.1 (60.8–94.9)	81.3 (61.8–100.9)	79.1 (60.8–97.3)	81.7 (65.5–100.2)	0.467
Current smoker, *n* (%)	19 (14.5)	6 (5.0)*	44 (17.1)*	21 (12.4)	0.012
Former smoker, *n* (%)	80 (61.1)*	86 (71.1)	198 (77.0)*	130 (76.5)	0.005
Diabetes mellitus, *n* (%)	25 (19.1)	18 (14.9)	46 (17.9)	35 (20.6)	0.653
Cardiovascular disease, *n* (%)	20 (15.3)	17 (14.0)	25 (9.7)	22 (12.9)	0.386
Osteoporosis, *n* (%)	7 (5.3)	5 (4.1)	24 (9.3)	13 (7.6)	0.243
Physical activity level (Mets hours/week)	25.0 (12.6–41.2)	41.6 (23.8–65.7)^†^	25.4 (15.7–43.8)^#^	44.9 (25.2–79.6)^†^ ^,^ ^§^	<0.001
Skeletal muscle index (kg/m^2^)	7.9 (7.4–8.5)	7.7 (7.1–8.3)	7.8 (7.3–8.5)	7.9 (7.4–8.5)	0.130
Handgrip strength (kg)	31.7 (27.8–35.8)	31.5 (27.6–34.1)	32.3 (28.3–36.0)	33.7 (29.9–37.0)^†^ ^,^ ^#^	0.003
Leg extension power (Nm/kg)	1.4 (1.2–1.6)	1.5 (1.1–1.8)	1.4 (1.2–1.7)	1.6 (1.2–1.8)^§^	0.021
Leg flexion power (Nm/kg)	0.7 (0.6–0.9)	0.7 (0.5–0.9)	0.7 (0.5–0.9)	0.8 (0.6–1.0)	0.114
Maximum gait speed (m/s)	1.9 (1.7–2.1)	1.9 (1.7–2.1)	1.9 (1.7–2.2)	2.0 (1.8–2.3)^†^	0.006
Myostatin (pg/mL)	2199.3 (1769.7–2775.9)	2026.8 (1673.2–2651.8)	2147.6 (1670.2–2743.9)	2125.8 (1667.8–2620.5)	0.714
BDNF (pg/mL)	19.2 (14.8–21.3)	19.9 (17.0–21.6)	18.7 (14.5–21.5)	19.3 (15.6–21.5)	0.374
Osteonectin (ng/mL)	282.4 (237.7–327.0)	282.8 (231.3–330.8)	277.6 (235.7–326.8)	271.2 (239.3–315.2)	0.865
FABP3 (pg/mL)	2697.0 (2024.0–3639.0)	2495.0 (1976.0–3430.0)	2685.0 (1934.5–3652.0)	2452.0 (1898.0–3315.3)	0.253
FGF‐21 (pg/mL)	296.1 (208.7–420.6)	234.5 (152.2–345.0)^†^	326.1 (210.4–521.3)^#^	270.0 (174.0–403.9)^§^	0.001

Women (*N* = 928)	None‐None (*N* = 297)	None‐Active (*N* = 196)	Active‐None (*N* = 264)	Active‐Active (*N* = 171)	*P* value
Age (years)	73 (69–78)	74 (69–78)	72 (68–77)	72 (69–77)	0.276
Height (cm)	152.2 (148.5–155.4)	152.0 (149.3–155.0)	153.0 (149.0–156.7)	153.1 (149.5–157.6)^†^	0.008
Body mass (kg)	51.3 (46.0–56.0)	50.7 (45.5–54.6)	51.4 (46.7–57.0)	51.7 (46.9–57.1)	0.165
Body mass index (kg/m^2^)	22.1 (20.0–24.5)	21.9 (19.9–23.7)	22.3 (20.3–24.3)	21.9 (20.0–24.2)	0.615
% body fat (%)	26.8 (23.1–30.3)	25.5 (21.9–29.2)	26.2 (23.0–29.6)	26.0 (22.3–28.7)	0.083
Fat‐free mass (kg)	37.1 (34.5–40.6)	36.8 (34.7–39.9)	37.5 (34.8–41.6)	38.3 (35.5–41.2)	0.034
Education years (years)	12.0 (12.0–14.0)	12.0 (12.0–15.0)	12.0 (12.0–14.0)	12.0 (12.0–14.0)	0.826
Protein intake (g/day)	78.3 (60.0–95.6)	78.1 (63.8–98.8)	75.1 (59.0–97.2)	78.6 (61.6–104.5)	0.700
Current smoker, *n* (%)	10 (3.4)	3 (1.5)	8 (3.0)	9 (5.3)	0.249
Former smoker, *n* (%)	49 (16.5)	24 (12.2)*	47 (17.8)	40 (23.4)*	0.043
Diabetes mellitus, *n* (%)	27 (9.1)	13 (6.6)	30 (11.4)	11 (6.4)	0.207
Cardiovascular disease, *n* (%)	22 (7.4)	9 (4.6)	17 (6.4)	10 (5.8)	0.645
Osteoporosis, *n* (%)	168 (56.6)	106 (54.1)	140 (53.0)	68 (39.8)*	0.004
Physical activity level (Mets hours/week)	24.6 (13.2–45.0)	34.7 (21.4–60.2)^†^	23.1 (13.0–43.1)^#^	39.1 (23.1–69.1)^†^ ^,^ ^§^	<0.001
Skeletal muscle index (kg/m^2^)	6.5 (5.9–7.0)	6.4 (6.0–7.0)	6.5 (6.0–7.0)	6.6 (6.1–7.0)	0.744
Handgrip strength (kg)	21.0 (18.6–23.4)	20.6 (19.2–22.8)	21.7 (19.3–23.7)	22.1 (19.9–24.5)^†^ ^,^ ^#^	<0.001
Leg extension power (Nm/kg)	1.2 (1.0–1.4)	1.2 (1.0–1.5)	1.2 (1.0–1.5)	1.3 (1.1–1.4)^†^	0.006
Leg flexion power (Nm/kg)	0.5 (0.4–0.7)	0.6 (0.4–0.7)	0.5 (0.4–0.7)	0.6 (0.5–0.7)^†^ ^,^ ^§^	0.002
Maximum gait speed (m/s)	1.8 (1.6–2.0)	1.8 (1.6–2.0)	1.8 (1.7–2.0)	1.9 (1.7–2.1)^†^	0.002
Myostatin (pg/mL)	1915.0 (1548.5–2328.1)	1836.2 (1489.6–2357.5)	1770.9 (1467.7–2171.3)	1775.6 (1523.2–2287.0)	0.091
BDNF (pg/mL)	18.7 (13.3–21.3)	19.2 (14.4–21.4)	19.3 (14.3–21.3)	19.1 (14.3–21.7)	0.391
Osteonectin (ng/mL)	266.3 (225.6–306.6)	273.5 (229.8–321.5)	271.9 (235.3–314.7)	279.7 (244.1–315.0)	0.122
FABP3 (pg/mL)	2473.0 (1854.0–3074.0)	2325.5 (1705.3–3041.3)	2158.5 (1735.3–2998.3)	2110.0 (1616.0–2684.0)^†^	0.011
FGF‐21 (pg/mL)	246.1 (168.2–352.7)	234.5 (164.0–325.4)	249.4 (175.7–374.9)	227.9 (150.4–358.0)	0.197

*Note*: Values are shown as median (interquartile range) for continuous variables and frequencies (percentage) for categorical variables.

Abbreviations: BDNF, brain‐derived neurotrophic factor; FABP3, fatty acid‐binding protein 3; FGF‐21, fibroblast growth factor 21; MET, metabolic equivalent.

^†^ *P* < 0.05 for significant differences compared to the None‐None group.

^#^ *P* < 0.05 for significant differences compared to the None‐Active group.

^§^ *P* < 0.05 for significant differences compared to the Active‐None group.

* *P* < 0.05 for significant difference among groups for the chi‐squared tests.

**Figure 2:**
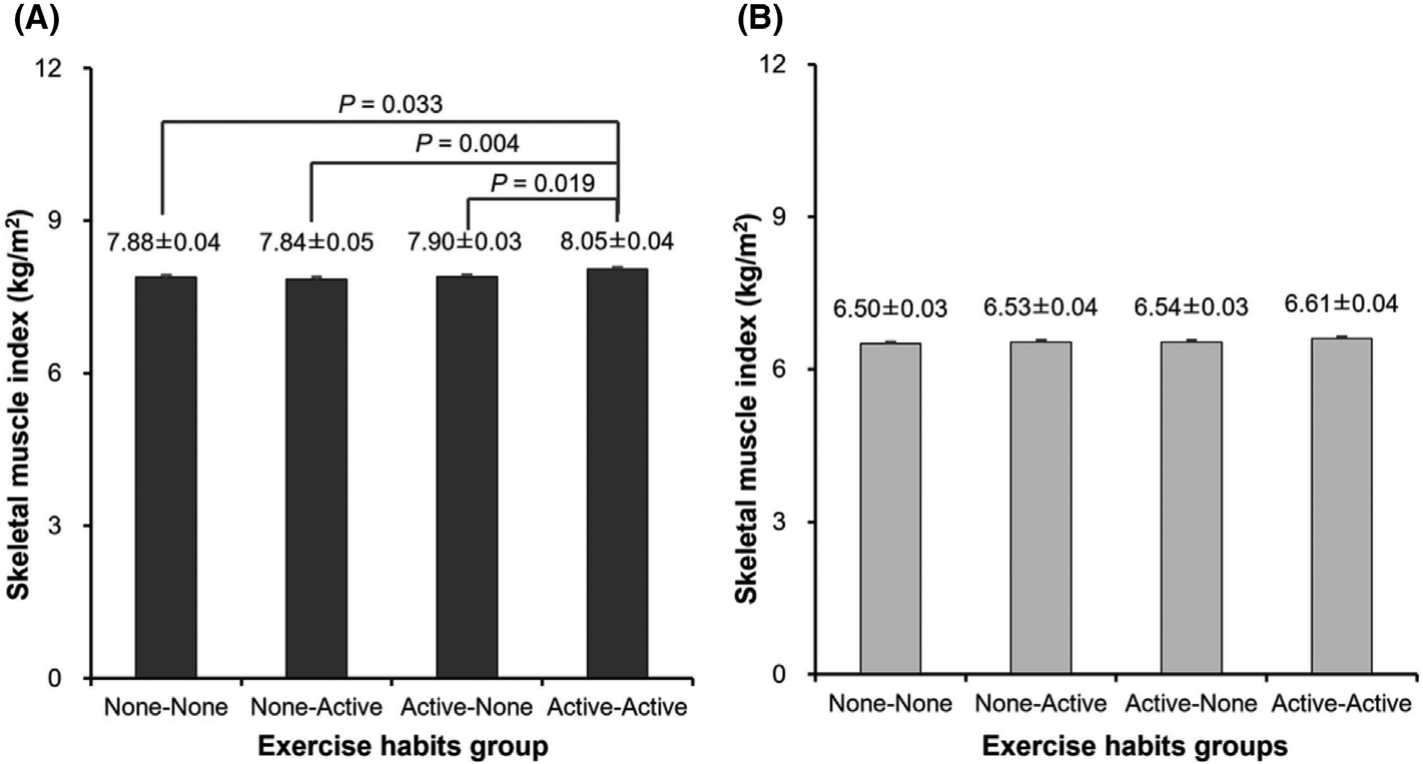
Comparison of skeletal muscle index among the four groups in men (A) and women (B). Values are presented as means ± SE. Adjusted variables: age, body mass index, education years, smoking habits, protein intake, current diabetes mellitus, current cardiovascular disease and current osteoporosis.

**Figure 3:**
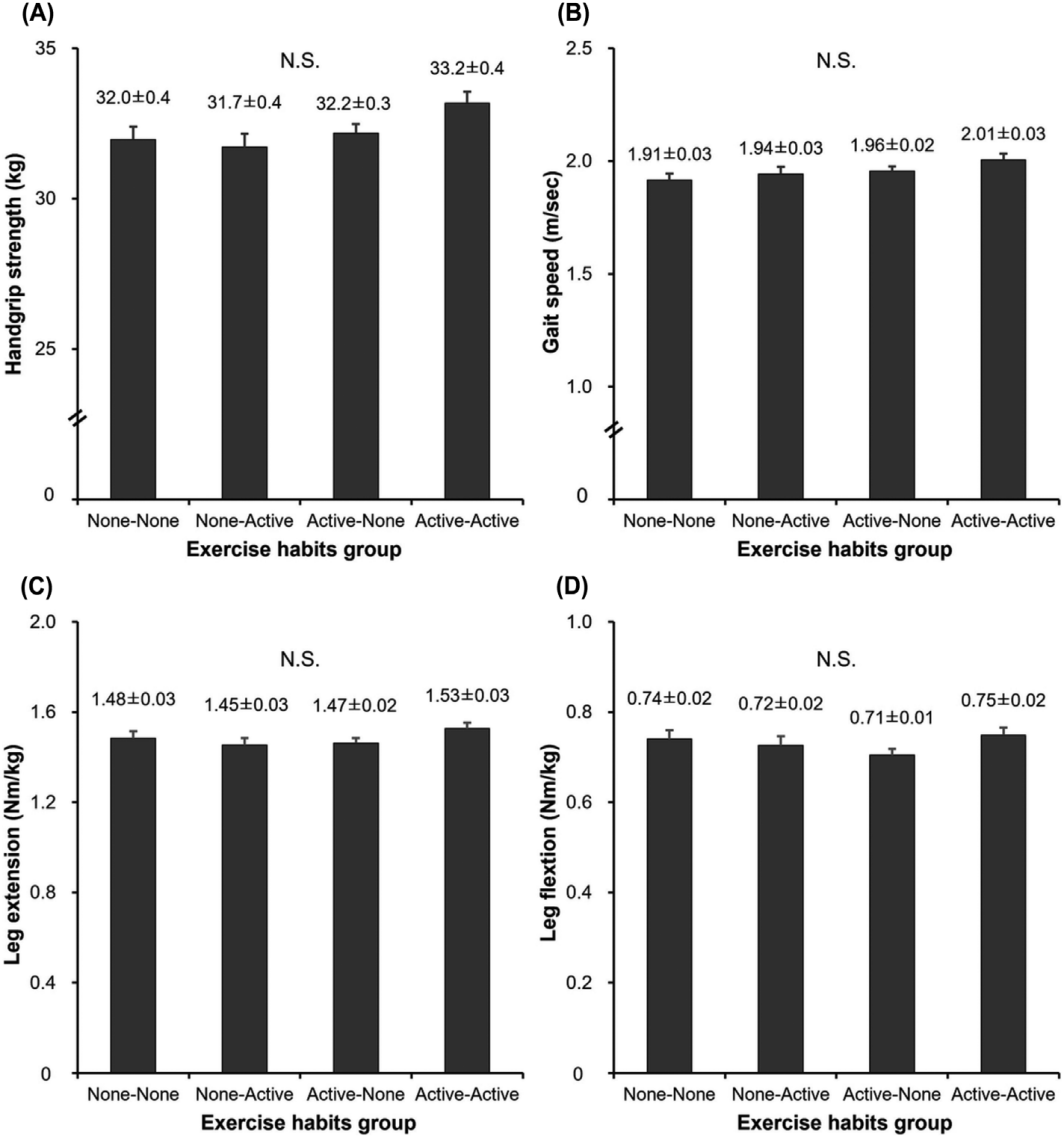
Comparison of the physical performance among four groups of men. Handgrip strength (A), gait speed (B), leg extension (C) and leg flexion (D). Values are presented as means ± SE. Adjusted variables: age, body mass index, education years, smoking habits, protein intake, current diabetes mellitus, current cardiovascular disease and current osteoporosis.

**Figure 4:**
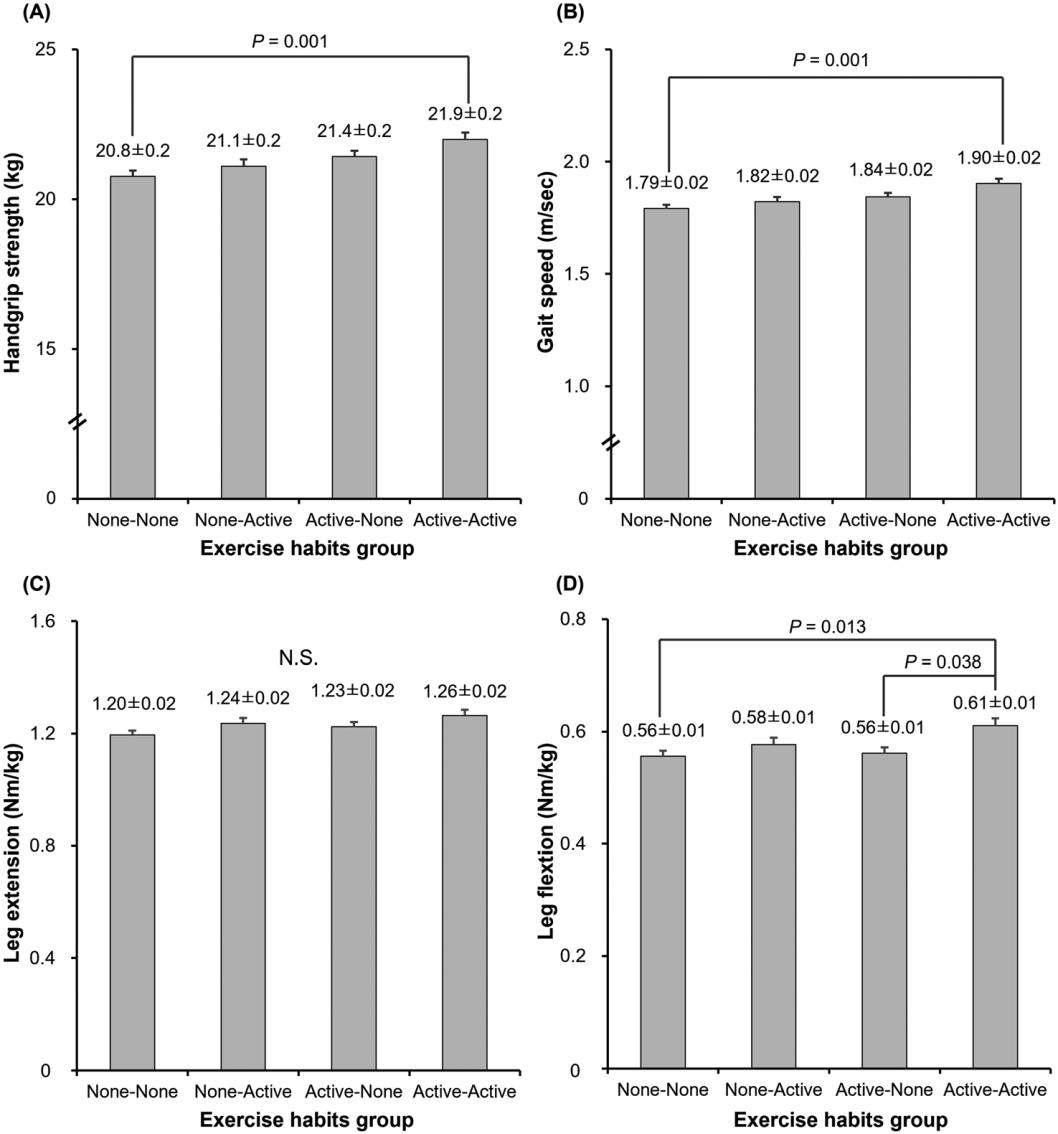
Comparison of the physical performance among four groups of women. Handgrip strength (A), gait speed (B), leg extension (C) and leg flexion (D). Values are presented as means ± SE. Adjusted variables: age, body mass index, education years, smoking habits, protein intake, current diabetes mellitus, current cardiovascular disease and current osteoporosis.

Differences in skeletal muscle function among the four groups were compared using ANCOVA adjusted for potential confounders (*Figures* 2, 3, 4). In men, SMI was significantly higher in the AA group than in the other three groups (vs. the NN group *P* = 0.033; vs. the NA group *P* = 0.004; vs. the AN group *P* = 0.019, respectively), whereas it was comparable among groups in women (*Figure* 2). In men (*Figure* 3), no significant differences were observed in handgrip strength, maximum gait speed, leg extension power, and leg flexion power among groups. In women (*Figure* 4), handgrip strength and gait speed were significantly higher in the AA group than in the NN group (*P* < 0.001) and tended to be higher than in the AN group (*P =* 0.070). Maximum gait speed was higher in the AA group than in the NN group (*P* = 0.001) and tended to be higher than in the NA group (*P =* 0.061). Leg extension power was not significant difference among the groups; however, it tended to be higher in the AA group than in the NN group (*P =* 0.065). Leg flexion power was significantly higher in the AA group than in the NN (*P =* 0.013) and AN groups (*P =* 0.038).

Myokine levels were compared among groups (*Table* 2). In men, no significant differences were observed in myostatin, BDNF, osteonectin or FABP3 concentrations among groups; however, FGF‐21 levels were significantly higher in the AN group than in the NA group (*P <* 0.001). In women, no significant differences were observed in myokine concentrations among groups.

**Table 2:** Comparisons of the myokine concentrations among groups

Men (*N* = 679)					
	None‐None (*N* = 131)	None‐Active (*N* = 121)	Active‐None (*N* = 257)	Active‐Active (*N* = 170)	*P* value
Myostatin (pg/mL)	2336.5 ± 80.6	2334.3 ± 83.2	2289.0 ± 56.9	2289.1 ± 70.1	0.940
BDNF (pg/mL)	18.1 ± 0.5	18.7 ± 0.5	17.7 ± 0.3	17.9 ± 0.4	0.328
Osteonectin (ng/mL)	289.8 ± 16.9	307.0 ± 17.5	301.2 ± 11.9	292.3 ± 14.7	0.865
FABP3 (pg/mL)	2968.2 ± 194.8	2866.2 ± 201.2	3264.1 ± 137.5	2903.0 ± 169.5	0.246
FGF‐21 (pg/mL)^a^	344.0 ± 21.3	275.9 ± 22.2	397.7 ± 15.3^#^	340.6 ± 18.7	<0.001

Women (*N* = 928)					
	None‐None (*N* = 297)	None‐Active (*N* = 196)	Active‐None (*N* = 264)	Active‐Active (*N* = 171)	*P* value
Myostatin (pg/mL)	2025.1 ± 38.1	1939.6 ± 47.0	1904.2 ± 40.4	1901.0 ± 50.6	0.106
BDNF (pg/mL)	17.2 ± 0.3	17.9 ± 0.4	17.9 ± 0.3	17.8 ± 0.4	0.393
Osteonectin (ng/mL)^b^	265.9 ± 83.4	501.8 ± 102.8	282.5 ± 88.4	318.2 ± 110.6	0.299
FABP3 (pg/mL)	2621.5 ± 77.8	2546.9 ± 96.0	2559.6 ± 82.6	2323.7 ± 103.3	0.141
FGF‐21 (pg/mL)^c^	275.0 ± 9.6	266.2 ± 11.9	294.9 ± 10.2	279.1 ± 12.9	0.292

*Note*: Values are presented as means ± standard errors. Adjusted variables: age, body mass index, education years, smoking habits, protein intake, current diabetes mellitus, current cardiovascular disease and current osteoporosis.

Abbreviations: BDNF, brain‐derived neurotrophic factor; FABP3, fatty acid‐binding protein 3; FGF‐21, fibroblast growth factor‐21.

^a^ FGF‐21 in men was analysed for 667 men (None‐None group 131; None‐Active group 119; Active‐None group 249; Active‐Active group 168) owing to missing data.

^b^ Osteonectin in women was analysed for 927 women (None‐None group 296; None‐Active group 196; Active‐None group.264; Active‐Active group 171) owing to missing data.

^c^ FGF‐21 in women was analysed for 920 women (None‐None group 297; None‐Active group 195; Active‐None group 263; Active‐Active group 165) owing to missing data.

^#^ *P* < 0.05 for significant differences compared to the None‐Active group.

### Association between the combination of exercise habits and ORs of skeletal muscle status

The total prevalence of sarcopenia was 6.6% (45/679) in men and 1.7% (16/928) in women, the prevalence of low muscle mass was 14.3% (97/679) in men and 5.2% (48/928) in women, and the prevalence of low muscle performance, which was defined as low muscle strength and/or low physical performance, was 25.6% (174/679) in men and 19.6% (182/928) in women.

The ORs for sarcopenia, low skeletal muscle mass and low muscle performance status in the NA, AN, and AA groups as compared to the NN group in men and women are listed in *Tables* 3 and 4, respectively. In men (*Table* 3), after covariate adjustment (Model 3), the ORs for sarcopenia, low muscle mass and low muscle performance were significantly lower in the AA group than in the NN group (sarcopenia; OR: 0.29, 95% CI: 0.09–0.95, *P* = 0.041, low muscle mass; OR: 0.21, 95% CI: 0.09–0.52, *P* = 0.001 and low muscle performance; OR: 0.52, 95% CI: 0.28–0.97, *P* = 0.038); the ORs for low muscle mass tended to be lower in the AN group than in the NN group (OR: 0.49, 95% CI: 0.23–1.04, *P* = 0.064). In women (*Table* 4), following covariate adjustment (Model 3), the ORs for low muscle performance were significantly lower in the AA group than in the NN group (OR: 0.48, 95% CI: 0.27–0.84 *P* = 0.010), whereas none of the ORs for sarcopenia and low muscle mass were significant.

**Table 3:** Association of the combination of exercise habits and the prevalence of sarcopenia and low muscle function in men

	Sample	Prevalence	Crude		Model 1		Model 2		Model 3		Model 4	
	*n*	*n* (%)	OR (95%CI)	*P* value	OR (95%CI)	*P* value	OR (95%CI)	*P* value	OR (95%CI)	*P* value	OR (95%CI)	*P* value
Sarcopenia												
None‐None	131	9 (6.9)	1.00		1.00		1.00		1.00		1.00	
None‐Active	121	11 (9.1)	1.36 (0.54–3.39)	0.516	0.91 (0.34–2.49)	0.860	0.95 (0.34–2.64)	0.915	0.97 (0.34–2.72)	0.950	0.99 (0.35–2.80)	0.983
Active‐None	257	19 (7.4)	1.08 (0.48–2.46)	0.851	0.78 (0.31–1.94)	0.595	0.81 (0.32–2.04)	0.651	0.80 (0.31–2.04)	0.635	0.74 (0.28–1.92)	0.532
Active‐Active	170	6 (3.5)	0.50 (0.17–1.43)	0.194	0.32 (0.10–1.03)	0.056	0.29 (0.09–0.97)	0.044	0.29 (0.09–0.95)	0.041	0.27 (0.08–0.91)	0.035
Low muscle mass												
None‐None	131	20 (15.3)	1.00		1.00		1.00		1.00		1.00	
None‐Active	121	23 (19.0)	1.30 (0.67–2.52)	0.431	0.66 (0.29–1.48)	0.309	0.67 (0.29–1.53)	0.342	0.70 (0.30–1.59)	0.388	0.69 (0.30–1.59)	0.388
Active‐None	257	37 (14.4)	0.93 (0.52–1.68)	0.819	0.51 (0.25–1.07)	0.073	0.52 (0.25–1.10)	0.086	0.49 (0.23–1.04)	0.064	0.48 (0.23–1.03)	0.059
Active‐Active	170	17 (10.0)	0.62 (0.31–1.23)	0.170	0.24 (0.10–0.58)	0.002	0.23 (0.10–0.57)	0.001	0.21 (0.09–0.52)	0.001	0.21 (0.08–0.52)	0.001
Low muscle performance^a^												
None‐None	131	39 (29.8)	1.00		1.00		1.00		1.00		1.00	
None‐Active	121	39 (32.2)	1.12 (0.66–1.91)	0.673	1.21 (0.68–2.15)	0.520	1.25 (0.69–2.24)	0.459	1.30 (0.72–2.35)	0.383	1.31 (0.73–2.38)	0.366
Active‐None	257	70 (27.2)	0.88 (0.55–1.40)	0.600	1.04 (0.63–1.71)	0.889	1.10 (0.66–1.82)	0.724	1.11 (0.66–1.85)	0.703	1.13 (0.67–1.91)	0.635
Active‐Active	170	26 (15.3)	0.43 (0.24–0.75)	0.003	0.53 (0.29–0.96)	0.035	0.54 (0.30–0.99)	0.047	0.52 (0.28–0.97)	0.038	0.54 (0.29–1.00)	0.050

*Note*: Model 1: adjusted for age and body mass index. Model 2: adjusted for model 1 covariates + education years, smoking habits and protein intake. Model 3: adjusted for model 2 covariates + current diabetes mellitus, current cardiovascular disease and current osteoporosis. Model 4: adjusted for model 3 covariates + exercise habits from young to middle age.

Abbreviations: CI, confidence interval; OR, odds ratio.

^a^ Low muscle performance defined as low muscle strength and/or low physical performance.

**Table 4:** Association of the combination of exercise habits and the prevalence of sarcopenia and low muscle function in women

	Sample	Prevalence	Crude		Model 1		Model 2		Model 3		Model 4	
	*n*	*n* (%)	OR (95%CI)	*P* value	OR (95%CI)	*P* value	OR (95%CI)	*P* value	OR (95%CI)	*P* value	OR (95%CI)	*P* value
Sarcopenia												
None‐None	297	4 (1.3)	1.00		1.00		1.00		1.00		1.00	
None‐Active	196	3 (1.5)	1.14 (0.25–5.14)	0.866	0.54 (0.09–3.36)	0.509	0.48 (0.07–3.17)	0.448	0.42 (0.06–3.06)	0.395	0.42 (0.06–3.21)	0.404
Active‐None	264	8 (3.0)	2.29 (0.68–7.69)	0.180	2.59 (0.62–10.77)	0.191	2.75 (0.63–11.99)	0.177	2.85 (0.62–13.06)	0.178	3.32 (0.70–15.86)	0.132
Active‐Active	171	1 (0.6)	0.43 (0.05–3.89)	0.453	0.15 (0.01–2.00)	0.153	0.15 (0.01–2.21)	0.168	0.06 (0.00–1.56)	0.091	0.07 (0.00–1.72)	0.103
Low muscle mass												
None‐None	297	12 (4.0)	1.00		1.00		1.00		1.00		1.00	
None‐Active	196	10 (5.1)	1.28 (0.54–3.02)	0.577	0.99 (0.35–2.83)	0.988	1.05 (0.36–3.06)	0.929	1.04 (0.36–3.06)	0.938	1.05 (0.36–3.08)	0.935
Active‐None	264	18 (6.8)	1.74 (0.82–3.68)	0.149	2.04 (0.84–4.97)	0.117	2.29 (0.92–5.74)	0.077	2.26 (0.89–5.70)	0.085	2.27 (0.88–5.81)	0.088
Active‐Active	171	8 (4.7)	1.17 (0.47–2.91)	0.743	0.98 (0.32–3.02)	0.974	1.10 (0.35–3.46)	0.875	0.92 (0.29–2.99)	0.895	0.93 (0.28–3.09)	0.906
Low muscle performance^a^												
None‐None	297	73 (24.6)	1.00		1.00		1.00		1.00		1.00	
None‐Active	196	37 (18.9)	0.71 (0.46–1.11)	0.138	0.64 (0.39–1.02)	0.062	0.63 (0.39–1.02)	0.059	0.65 (0.40–1.05)	0.081	0.68 (0.42–1.11)	0.124
Active‐None	264	50 (18.9)	0.72 (0.48–1.08)	0.108	0.73 (0.47–1.13)	0.159	0.72 (0.47–1.12)	0.147	0.70 (0.45–1.08)	0.109	0.74 (0.47–1.16)	0.185
Active‐Active	171	22 (12.9)	0.45 (0.27–0.76)	0.003	0.45 (0.26–0.78)	0.005	0.45 (0.26–0.79)	0.005	0.48 (0.27–0.84)	0.010	0.53 (0.30–0.94)	0.031

*Note*: Model 1: adjusted for age and body mass index. Model 2: adjusted for model 1 covariates + education years, smoking habits and protein intake. Model 3: adjusted for model 2 covariates + current diabetes mellitus, current cardiovascular disease and current osteoporosis. Model 4: adjusted for model 3 covariates + exercise habits from young to middle age.

Abbreviations: CI, confidence interval; OR, odds ratio.

^a^ Low muscle performance defined as low muscle strength and/or low physical performance.

Additionally, we examined preliminary adjusting for the young adult to middle‐age exercise habits score (Model 4). In men (*Table* 3), the ORs for sarcopenia and low muscle mass remained significantly lower in the AA group than in the NN group (sarcopenia; OR: 0.27, 95% CI: 0.08–0.91 *P* = 0.041, low muscle mass; OR: 0.21, 95% CI: 0.08–0.52, *P* = 0.001); low muscle performance difference was attenuated but tended to be lower in the AA group than in the NN group (OR: 0.54, 95% CI: 0.29–1.00, *P* = 0.0502). Also, in women (*Table* 4), the ORs for low muscle performance difference attenuated but remained significantly lower in the AA group (OR: 0.53, 95% CI: 0.30–0.94 *P* = 0.031).

## Discussion

In the present study, we investigated the associations between the combination of exercise habits in adolescence and older age with sarcopenia and its components in community‐dwelling Japanese older adults. The ANCOVA analysis revealed that the AA group showed higher physical functionality, including in regard to hand grip strength, gait speed and leg muscle strength than the NN group in women, while these measures were comparable across groups in men. On the other hand, the SMI in the AA group was higher than in the other three groups in men, while the SMI was comparable among the four study groups in women. Similarly, in women, the OR for low muscle performance was lower in the AA group than in the NN group, whereas the ORs for low muscle mass in each group were not significant. In men, the ORs for low muscle mass and muscle performance were lower in the AA group than in the NN group. The OR for sarcopenia was significantly lower in the AA group in men and comparable among groups in women.

In our study, older men with exercise habits in both adolescence and older age had a lower prevalence of sarcopenia, and older women with exercise habits in both adolescence and older age showed higher muscle performance. These results support our hypothesis that combined exercise habits in adolescence and older age are most effective for maintaining high muscle function in older age, as muscle function reaches its peak in one's 20s and gradually decreases after one's 50s.^12^ In addition, previous studies have reported that individuals with past exercise habits have greater training effects for retraining after subsequent de‐training compared with those without past exercise habits.^19^ Thus, it may be beneficial for older adults to implement exercise habits in both adolescence and old age to maintain muscle function.

There may be sex differences in reference to the combined effects of exercise habits in adolescence and older age. In the current study, men in the AA group had a higher muscle mass, whereas there were no differences in muscle mass among groups defined according to exercise habits in women (*Figure* 2). A previous study reported a greater decline in muscle mass with ageing in men than in women.^27^ Therefore, findings to date suggest that a difference in muscle mass may be more likely to appear in men than in women. On the other hand, regardless of the lack of difference in muscle mass across exercise groups in women, the AA group showed higher muscle performance than the NN and NA groups (*Figure* 4). In men, muscle performance was comparable among the study groups, despite the higher muscle mass evidenced in the AA group (*Figure* 3). Accordingly, it has been reported that the decline in muscle strength seen with ageing is not necessarily dependent on the loss of muscle mass.^28^ However, the underlying reasons why associations among exercise habits, muscle volume and muscle performance in men are reported to be opposite to those detected in women, including in this study, remain unclear.

In the present study, the prevalence of sarcopenia was 6.6% in men and 1.7% in women. These prevalence values were lower than the prevalence of sarcopenia (approximately 5–20%) defined by AWGS 2019, according to the findings of previous studies conducted in community‐dwelling Japanese older adults.^29^, ^30^ While these previous studies used bioimpedance analysis to measure body composition, we used DXA in our current research. This may be one reason for the low prevalence of sarcopenia detected in the present study, as the AWGS criteria define different cut‐off values for SMI for bioimpedance and DXA. Additionally, the prevalence of sarcopenia may also vary by target cohort population. Our cohort included only those living in Bunkyo‐Ku, Tokyo, an urban area in Japan, while other studies included various other cities and prefectures (i.e. Kusatsu City in Gunma Prefecture, Hatoyama Town in Saitama Prefecture, and Obu City in Aichi Prefecture).^29^, ^30^

Similar to our study, a previous study reported that older adults with exercise habits in early adulthood and older age showed a higher appendicular lean mass and handgrip strength than those who did not exercise in both periods.^31^ However, this previous study evaluated exercise habits in early adulthood, before age 30, rather than in adolescence; thus, the contribution of exercise habits in adolescence towards muscle function and sarcopenia was previously unknown and, to our knowledge, was firstly shown in the present study. We note that, in Japan, the exercise implementation rate has been shown to be the highest during junior high school (73.7% in males and 57.0% in females)^32^ and that exercise rates subsequently massively decrease with age, reaching a steady level of 40–50% by the 20–50 year age range.^33^ Therefore, exercise habits during adolescence may play an important role in the prevention of sarcopenia in older Japanese adults.

In terms of myokines, only the level FGF‐21 was lower in the NA group than in the AN group in men, while the levels of other myokines were similar among the study groups, and there were also no differences in women. A previous report showed that moderate‐to‐vigorous‐intensity physical activity was negatively associated with circulating FGF‐21 levels.^34^ Consistently, we found that physical activity levels were higher in the NA group than in the AN group. Additionally, the FGF‐21 level has been shown to be increased by smoking,^35^ and we speculate that differences in smoking rates across study groups may have caused intergroup differences in circulating FGF‐21 levels. We observed a trend towards higher smoking rates in the AN group and lower smoking rates in the NA group.

The current study has several limitations. First, there may have been recall bias, as we collected data on exercise habits via a self‐reported questionnaire. Additionally, we did not consider some specific information in regard to exercise, including types, quantities, intensities and frequencies of exercise. In Japanese junior and senior high schools, school‐based sports club activities termed ‘Bukatsudo’ is organized as a part of the educational curriculum.^36^ Participating with other students in school‐based sports clubs could be relatively memorable. Therefore, to minimize recall bias and ensure the validity of the questionnaires, we defined exercise habits in adolescents according to participation in sports club activities in junior high school and high school. Second, the present study did not consider exercise habits in the period between adolescence and older age. At least our preliminary analyses showed group differences in exercise habits in the period between adolescence and old age (*Figure* S1), but these differences did not significantly affect the results of the logistic regression analysis (*Tables* 3 and 4). However, as mentioned above, our exercise habits data in the period between adolescence and old age appear to have greater recall bias than in adolescence or old age. Therefore, a future long‐term prospective cohort study that followed the participants from adolescence to old age is needed to confirm these preliminary data. Third, this study was cross‐sectional, and we were therefore not able to draw causal inferences. Fourth, owing to the low prevalence of sarcopenia in women, the OR for sarcopenia may not have been adequately determined in women in the current study. Hence, future studies were needed a longitudinal design study and replication study in other cohorts.

In conclusion, we found that older men with exercise habits in both adolescence and older age were at a lower risk of sarcopenia, low muscle mass and low muscle performance, whereas older women with exercise habits in both adolescence and older age were at a lower risk of low muscle performance. A combination of exercise habits in adolescence and older age may therefore be effective for maintaining optimal muscle function with ageing.

## Conflict of interest

Y.T. and R.K. received research support from Curves Japan Co., Ltd. Y.T. received research support from LOTTE Co., Ltd. and Imasen Electric Industrial Co., Ltd. These funders had no role in the current research. The other authors have no actual or potential conflicts of interest to declare.

## Supporting information


**Figure S1.** Exercise participation rate (%) by four exercise groups in men (a) and women (b). ^†^ *P* < 0.05 for significant differences compared to the None‐None group, ^#^ *P* < 0.05 for significant differences compared to the None‐Active group, ^§^ *P* < 0.05 for significant differences compared to the Active‐None group for the Chi‐squared tests.

**Table S1.** The cumulate number of people for each sport in adolescence exercise habits, called “Bukatsudo”.
**Table S2.** The cumulate number of people for each sport in current exercise habits.
