# Supplementary figures and images for: Effects of exercise habits in adolescence and older age on sarcopenia risk in older adults: the Bunkyo Health Study
Source: J Cachexia Sarcopenia Muscle. 2023 Apr 13;14(3):1299–311. doi: 10.1002/jcsm.13218 (PMC10235900; doi:10.1002/jcsm.13218)

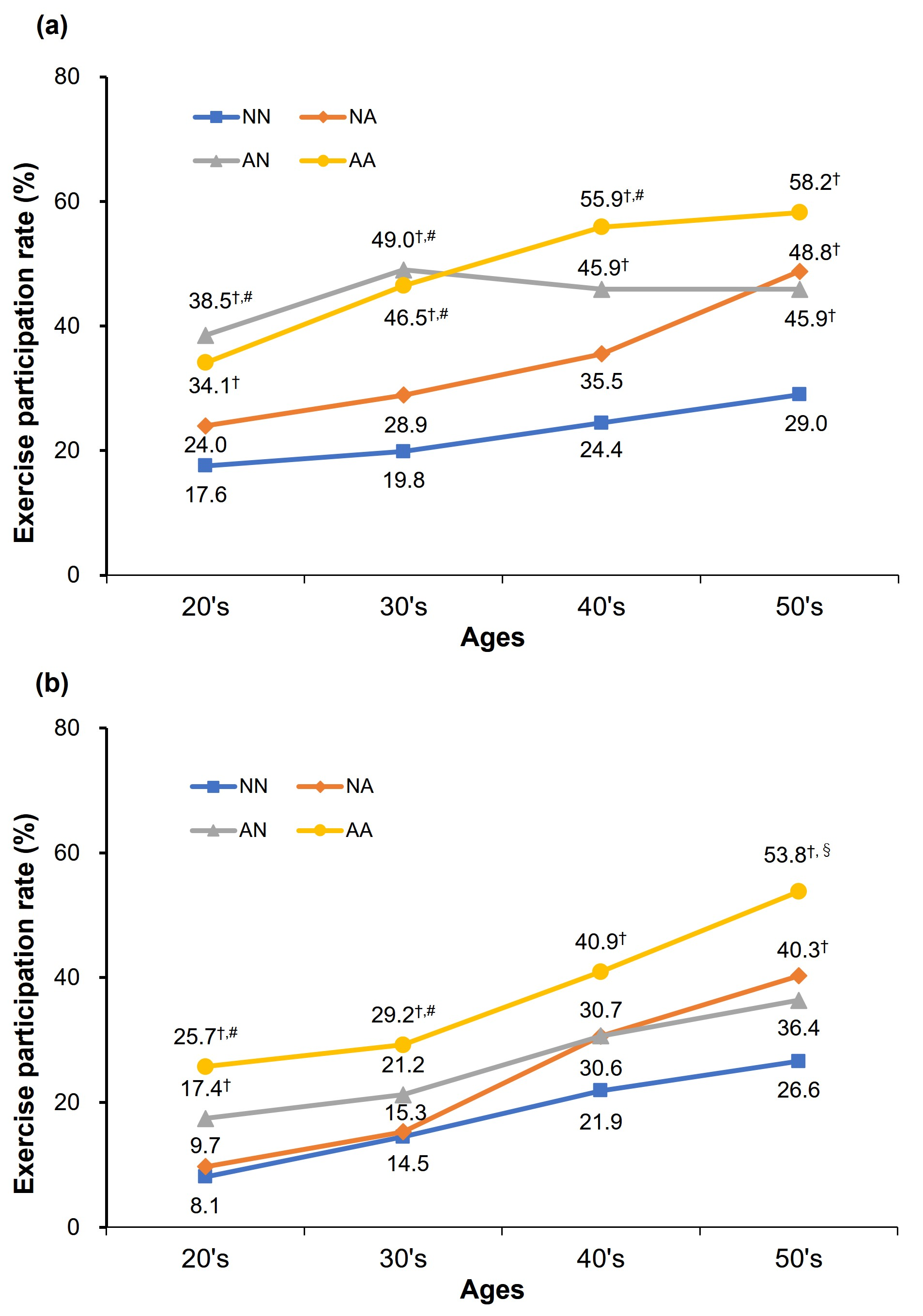

Supplement: Supplementary file 1 — Figure S1. Exercise participation rate (%) by four exercise groups in men (a) and women (b). † P < 0.05 for significant differences compared to the None‐None group, # P < 0.05 for significant differences compared to the None‐Active group, § P < 0.05 for significant differences compared to the Active‐None group for the Chi‐squared tests. [file JCSM-14-1299-s001.tif]
