# Supplementary material for: Effects of exercise habits in adolescence and older age on sarcopenia risk in older adults: the Bunkyo Health Study
Source: J Cachexia Sarcopenia Muscle. 2023 Apr 13;14(3):1299–311. doi: 10.1002/jcsm.13218 (PMC10235900; doi:10.1002/jcsm.13218)
Supplement: Supplementary file 2 — Table S1. The cumulate number of people for each sport in adolescence exercise habits, called “Bukatsudo”. Table S2. The cumulate number of people for each sport in current exercise habits. [file JCSM-14-1299-s002.docx]

**Supplementary Table S1.** The cumulate number of people for each sport in adolescence exercise habits, called "Bukatsudo."

|  | All (n=1068) | | Men (n=540) | | Women (n=528) | |
| --- | --- | --- | --- | --- | --- | --- |
|  | n | % | n | % | n | % |
| Volleyball | 189 | (17.7) | 37 | (6.9) | 152 | (28.8) |
| Baseball or Softball | 137 | (12.8) | 99 | (18.3) | 38 | (7.2) |
| Tennis | 134 | (12.5) | 45 | (8.3) | 89 | (16.9) |
| Basketball | 119 | (11.1) | 65 | (12.0) | 54 | (10.2) |
| Table tennis | 118 | (11.0) | 46 | (8.5) | 72 | (13.6) |
| Track & Field | 71 | (6.6) | 41 | (7.6) | 30 | (5.7) |
| Judo | 49 | (4.6) | 49 | (9.1) | 0 | (0.0) |
| Swimming | 45 | (4.2) | 29 | (5.4) | 16 | (3.0) |
| Gymnatics | 43 | (4.0) | 11 | (2.0) | 32 | (6.1) |
| Mountaineering | 36 | (3.4) | 20 | (3.7) | 16 | (3.0) |
| Soccer | 22 | (2.1) | 22 | (4.1) | 0 | (0.0) |
| Kendo | 18 | (1.7) | 15 | (2.8) | 3 | (0.6) |
| Ski and Skating | 14 | (1.3) | 8 | (1.5) | 6 | (1.1) |
| Dance | 13 | (1.2) | 0 | (0.0) | 13 | (2.5) |
| Rugby | 12 | (1.1) | 12 | (2.2) | 0 | (0.0) |
| Badminton | 7 | (0.7) | 3 | (0.6) | 4 | (0.8) |
| Kyudo | 6 | (0.6) | 5 | (0.9) | 1 | (0.2) |
| Boxing | 6 | (0.6) | 6 | (1.1) | 0 | (0.0) |
| Rowing and Sailing | 5 | (0.5) | 4 | (0.7) | 1 | (0.2) |
| Ice hockey | 4 | (0.4) | 4 | (0.7) | 0 | (0.0) |
| Karate | 4 | (0.4) | 4 | (0.7) | 0 | (0.0) |
| Handball | 4 | (0.4) | 4 | (0.7) | 0 | (0.0) |
| Wrestling | 4 | (0.4) | 4 | (0.7) | 0 | (0.0) |
| Golf | 3 | (0.3) | 2 | (0.4) | 1 | (0.2) |
| Equestrian art | 2 | (0.2) | 2 | (0.4) | 0 | (0.0) |
| American football | 1 | (0.1) | 1 | (0.2) | 0 | (0.0) |
| Aviation | 1 | (0.1) | 1 | (0.2) | 0 | (0.0) |
| Weightlifting | 1 | (0.1) | 1 | (0.2) | 0 | (0.0) |

**Supplementary Table S2.** The cumulate number of people for each sport in current exercise habits.

|  | All (n=1441) | | Men (n=578) | | Women (n=863) | |
| --- | --- | --- | --- | --- | --- | --- |
|  | n | % | n | % | n | % |
| Walking | 285 | (19.8) | 163 | (28.2) | 122 | (14.1) |
| Calisthenics | 190 | (13.2) | 38 | (6.6) | 152 | (17.6) |
| Golf | 110 | (7.6) | 87 | (15.1) | 23 | (2.7) |
| Resistance training | 106 | (7.4) | 55 | (9.5) | 51 | (5.9) |
| Health club activities | 105 | (7.3) | 26 | (4.5) | 79 | (9.2) |
| Stretching | 91 | (6.3) | 21 | (3.6) | 70 | (8.1) |
| Swimming | 78 | (5.4) | 29 | (5.0) | 49 | (5.7) |
| Yoga and Pilates | 63 | (4.4) | 6 | (1.0) | 57 | (6.6) |
| Aquabics | 62 | (4.3) | 20 | (3.5) | 42 | (4.9) |
| Tennis | 61 | (4.2) | 29 | (5.0) | 32 | (3.7) |
| Aerobic gymnastics | 55 | (3.8) | 8 | (1.4) | 47 | (5.4) |
| Social dance | 46 | (3.2) | 7 | (1.2) | 39 | (4.5) |
| Running | 41 | (2.8) | 31 | (5.4) | 10 | (1.2) |
| Tai Chi and Qi gong | 32 | (2.2) | 1 | (0.2) | 31 | (3.6) |
| Table tennis | 23 | (1.6) | 7 | (1.2) | 16 | (1.9) |
| Cycling exercise | 18 | (1.2) | 8 | (1.4) | 10 | (1.2) |
| Mountaineering | 15 | (1.0) | 10 | (1.7) | 5 | (0.6) |
| Volleyball | 10 | (0.7) | 2 | (0.3) | 8 | (0.9) |
| Budo | 5 | (0.3) | 3 | (0.5) | 2 | (0.2) |
| Table top curling | 5 | (0.3) | 1 | (0.2) | 4 | (0.5) |
| Ski | 5 | (0.3) | 2 | (0.3) | 3 | (0.3) |
| Badminton | 5 | (0.3) | 3 | (0.5) | 2 | (0.2) |
| Baseball | 4 | (0.3) | 4 | (0.7) | 0 | (0.0) |
| Boxing and Boxercise | 4 | (0.3) | 3 | (0.5) | 1 | (0.1) |
| Basketball | 4 | (0.3) | 4 | (0.7) | 0 | (0.0) |
| Ballet | 3 | (0.2) | 0 | (0.0) | 3 | (0.3) |
| Shooting and Darts | 3 | (0.2) | 2 | (0.3) | 1 | (0.1) |
| Bowling | 3 | (0.2) | 2 | (0.3) | 1 | (0.1) |
| Rowing and Sailing | 3 | (0.2) | 3 | (0.5) | 0 | (0.0) |
| Kyudo | 2 | (0.1) | 0 | (0.0) | 2 | (0.2) |
| Horse riding | 1 | (0.1) | 1 | (0.2) | 0 | (0.0) |
| Ice hockey | 1 | (0.1) | 1 | (0.2) | 0 | (0.0) |
| Japanese croquet | 1 | (0.1) | 0 | (0.0) | 1 | (0.1) |
| Dodgeball | 1 | (0.1) | 1 | (0.2) | 0 | (0.0) |
